# A new approach in acute myeloid leukemia (AML): Samatya-predicting score

**DOI:** 10.1016/j.lrr.2022.100293

**Published:** 2022-02-15

**Authors:** Istemi Serin, Bagnu Orhan, Derya Sonmez, Tahir Alper Cinli, Hasan Goze, Huriye Serin, Begum Gulesir, Osman Yokus

**Affiliations:** a Department of Hematology, University of Health Sciences, Istanbul Training and Research Hospital, Org. Nafiz GURMAN Cad., Samatya, Fatih, Istanbul, Istanbul, 34098 Turkey; b Department of Medical Biochemistry, University of Health Sciences, Istanbul Training and Research Hospital, Istanbul, Turkey; c Department of Internal Medicine, University of Health Sciences, Istanbul Training and Research Hospital, Istanbul, Turkey

**Keywords:** Acute myeloid leukemia, Flow cytometry, Response, Prognosis

## Abstract

We aimed to examine the efficiency for prediction of prognosis and response in non-APL AML cases of the “Samatya-predicting score”. A total of 213 patients diagnosed between January 2010-December 2020 were examined. Of the 158 patients included in the study, the median value of risk score was determined as 2,5. The sensitivity for mortality was 88% and the specificity was 42%. In terms of being non-responder to induction therapy, the sensitivity was 90,1%, the specificity was 25,3%. OS was shorter in those with high risk scores. This study makes an important contribution to the literature in terms of creating a different perspective to predict prognosis in AML.

## Highlights

• We aimed to examine the efficiency of our new scoring system in non-APL AML cases,.
• The AUC for the median risk score of 2,5 in ROC analysis was 0,635 (*p* = 0,006) for exitus; 0,605 (*p* = 0,024) for being responder,.
• The sensitivity for mortality was 88%, the specificity 42%, the PPV 90,1%, and the NPV was 24,7%,.
• In terms of being non-responder to induction therapy, the sensitivity was 90,1%, the specificity 25,3%, the PPV 89,8%, and the NPV was 32%.

## Abbreviations

**AML**: Acute myeloid leukemia
**WHO**: World Health Organization
**Non-APL AML**: Non-acute promyelocytic leukemia
**CR**: Complete response
**CNS**: Central nervous system
**AUC**: The area under the curve
**PPV**: Positive predictive value
**NPV**: Negative predictive value
**OS**: Overall survival
**FLT3/ITD**: Fms-like tyrosine kinase-3 internal tandem duplication
**EFS**: Event free survival

## Background

Acute myeloid leukemia (AML) is a heterogeneous hematological malignancy characterized by clonal expansion of myeloid blasts in peripheral blood, bone marrow and/or other tissues [1]. It is the most common form of acute leukemia among adults. The gold standard method in AML classification is the World Health Organization (WHO) classification [2]. It is difficult to combine different parameters in order to obtain the most accurate result in evaluating prognosis in AML.

In addition to clinical, morphological and immunophenotyping evaluation, molecular cytogenetic methods are the main ones used in AML classification and predicting prognosis [2, 3]. The risk stratification of non-acute promyelocytic leukemia (non-APL) AML [3] consists of a 3-group classification system, and this way, it predicts prognosis while guiding treatment: Favorable, intermediate, poor/adverse. In a review of adult patients with AML treated on Cancer and Leukemia Group B protocols, the 5-year survival rates for patients with favorable, intermediate, and poor-risk subgroups were 55%, 24%, and 5%, respectively [4]. The AML 11 trial had similar 5-year survival rates of the favorable, intermediate, and poor-risk subgroups of 34%, 13%, and 2%, respectively [5].

Immunophenotypic characteristics are also markers that associated with prognosis. The clear association of cell surface markers with prognosis constitutes an important area of ​​study. In some studies, CD34 positivity has been shown to be associated with poor prognosis [6]. CD34 and HLA-DR........... coexistence were also found to be independent prognostic factors for induction failure [6], [7], [8]. Aberrant expression CD7 has been found to be positive prognostic indicator in AML [9]. New studies are needed in order to use all these different evaluation methods easily and effectively to predict the prognosis.

In our study, we aimed to examine the efficiency for prediction of prognosis and response to induction therapy in non-APL AML cases of the “Samatya-predicting score” that we developed using clinical, cytogenetic and flow cytometric features.

## Material and methods

A total of 213 patients diagnosed with non-APL AML between January 2010 and December 2020 were included in the study. Demographic data (age, gender), initial leukocyte counts, and other risk scoring system parameters were recorded. The patients' final status (exitus, alive) and their response to induction therapy (complete, incomplete, failure) were recorded. In the statistical analysis, the groups “responder” and “non-responder” were formed as follows: Responder: Complete response (CR); non-responder: Incomplete or failure.

## Inclusion- Exclusion Criteria

**Fig. 1:**
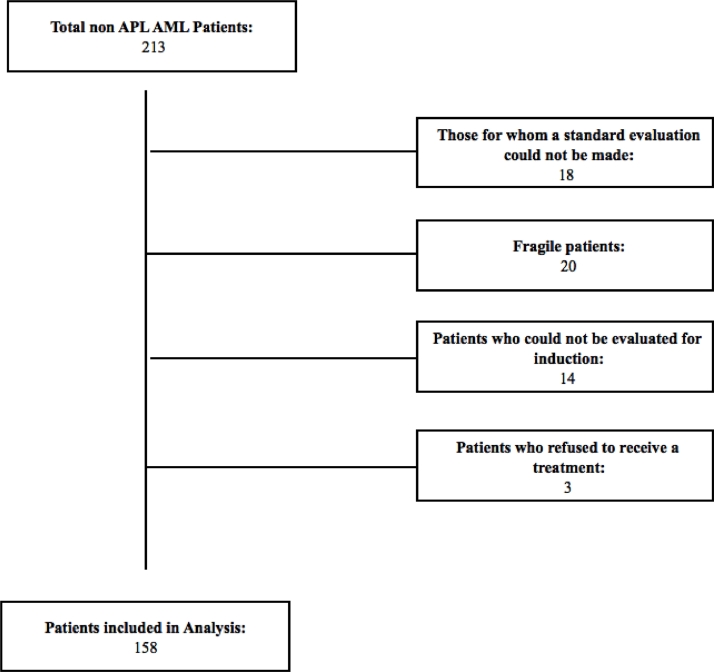
Patients’ flow-chart.

Out of a total of 213 patients, patients who were not eligible for the study were excluded (Fig. 1. Patients’ flow-chart). All patients received “7 + 3″ treatment: 7 + 3 treatment: 1.5–3 gr/m2 Cytarabine, every 12 h, D1–7; 12 mg/m2 idarubicin, D1–3 [10].

1. Those for whom a standard evaluation could not be made: Patients who did not / could not perform the basic examinations required for the risk scoring system such as bone marrow biopsy, flow cytometric examination, diagnosis chromosome analysis or cytogenetic examination,

2. Fragile patients: Patients who are not suitable for 7 + 3 treatment due to their comorbidities and fragility; patients who have been treated with different treatment options,

3. Patients who could not be evaluated for induction: Patients who died before the evaluation of response to induction therapy or who did not receive any induction therapy,

4. Patients who refused to receive 7 + 3 treatment were excluded from the study.

## Response to induction therapy

The patients' response to induction therapy was evaluated by bone marrow biopsy performed in 21–28 days after the start of treatment. With flow cytometric and pathological examination, blast count <5% was considered as CR, 5–20% as incomplete response and ≥20% as failure. If the total cellularity was less than 20%, recovery was expected by considering hypoplasia, and the response was confirmed by repeating the bone marrow biopsy [10].

## Risk scoring system

Risk scoring system parameters are shown in Table 1. Initial leukocyte count (<50,000 = 0, ≥50,000 = 1), age (<60 = 0, ≥60 = 1), central nervous system (CNS) involvement ((-) = 0, (+) = 1), presence of extramedullary disease ((-) = 0, (+) = 1), presence of tumor lysis during induction therapy ((-) = 0, (+) = 1), risk stratification by genetics in non-APL AML [3] (favorable = 0, intermediate = 1, poor/adverse = 2) and flow cytometry findings (CD7, CD34, HLA-DR........... Positivity = 1) were recorded and the total score was calculated.

**Table 1:** Risk scoring system parameters.

**Age**	
	<60 = 0
	≥60 = 1
Initial leukocyte count (/mm^3^)	
	<50.000 = 0
	≥50.000 = 1
CNS involvement	
	(-) = 0
	(+) = 1
Extramedullary disease	
	(-) = 0
	(+) = 1
Presence of tumor lysis	
	(-) = 0
	(+) = 1
**Risk stratification by genetic in non-APL AML**	
	Favorable = 0
	Intermediate = 1
	Poor = 2
Flow cytometry findings	
	CD7 Positivity = 1
	CD34 Positivity = 1
	HLA-DR........... Positivity = 1

**CNS.:** Central nervous system**, non-APL AML:** Non-acute promyelocytic leukemia.

For major neurologic signs or symptoms at diagnosis, appropriate imaging studies were performed to detect meningeal disease, chloromas, or CNS bleeding. Lumbar puncture (LP) was performed if no mass lesion is detected on the imaging study with central shift making an LP relatively contraindicated; and the sample was examined flow cytometrically and pathologically. Cerebrospinal fluid (CSF) analysis was routinely performed in all patients with AML with monocytic differentiation, extramedullary involvement, and >40,000 leukocytes at diagnosis [10].

## Laboratory analysis

Blood samples were taken from the patients in an EDTA tube. Samples were run on the XN 9000 (Sysmex, Kobe, Japan) instrument within 2 h. Flow cytometric analysis was performed on a 3-laser, 10-color FACS Lyric flow cytometry analyzer (BD Biosciences, San Jose, USA). Calibration control and compensation adjustment were made before the samples were run. The performance of the system was checked by passing standardized beads. Samples taken into EDTA tubes were kept at the room temperature and studied within 24 h. The fluorochromes and clones of the antibodies we used are CD7 APC (M-T701 clone), CD34 PERCP (8G12 clone), HLAR DR........... V450 (L243 clone), respectively. BD FACSSUITE Software was used for analysis.

## Statistical analysis

The analysis of the data was done with SPSS 26 program and it was studied with 95% confidence level. Frequency (n) and percentage (%) statistics are given for categorical (qualitative) variables, mean (X), standard deviation (SD), minimum and maximum statistics are given for numerical (quantitative) variables.

In the study, the relationship between mortality, response to induction therapy and grouped variables was analyzed with the Chi-square test. Independent groups *t*-test was used to compare mortality and response status according to OS, risk score. ROC analysis was used for the median values ​​of the risk score to predict mortality, response to induction therapy, and probability. Kaplan Meier analysis was used for survival analysis and curves. Chi-square test is a test technique used to determine the relationship between two categorical variables. Independent groups *t*-test is a test technique used to compare two groups in terms of a numerical measurement. ROC analysis consists of the test techniques in which the relevant disease/outcome variable is estimated according to the cut-off values ​​of the measurements in diagnostic tests. Sensitivity (the rate of detecting mortality, response to induction therapy), specificity (detection rate of survival, response rate), positive predictive (rate of positive value of the measurement being exitus, non-responder), negative predictive (rate of negative value of the measurement being alive, responder) probabilities were calculated. Kaplan Meier analysis is a test technique in which the factor affecting survival is examined in certain periods.

## Results

Of the 158 patients included in the study, 71 (44.9%) were female and 87 (55.1%) were male. The median age of the patients included in the study was 55 years (range: 18–88). Sixty-two of them were diagnosed below the age of 60; 68.4% had leukocyte count less than 50,000 at diagnosis, 6.3% had CNS involvement, 21.5% had extramedullary disease, and 0.6% had tumor lysis. According to the risk stratification by genetics in non APL-AML, 17.7% had poor genetic risk **(**Table 2**)**.

**Table 2:** Distribution of demographic characteristics of the patients.

		**n (%)**
**Gender**	Female	71 (44,9)
	Male	87 (55,1)
**Age**	<60	98 (62)
	≥60	60 (38)
	Min-Max	18–88
	Median	55
	Mean±*s*.d.	53,78±16,95
**Initial leukocyte count (/mm^3^)**	<50.000	108 (68,4)
	≥50.000	50 (31,6)
**CNS involvement**	(-)	148 (93,7)
	(+)	10 (6,3)
**Extramedullary disease**	(-)	124 (78,5)
	(+)	34 (21,5)
**Presence of tumor lysis**	(-)	157 (99,4)
	(+)	1 (0,6)
**Risk stratification by genetic in non-APL AML**	Favorable	66 (41,8)
	Intermediate	64 (40,5)
	Poor	28 (17,7)
**CD7**	(-)	79 (50)
	(+)	79 (50)
**CD34**	(-)	55 (34,8)
	(+)	103 (65,2)
**HLA DR...........**	(-)	21 (13,3)
	(+)	137 (86,7)
**Response to induction therapy**	Complete	87 (55,1)
	Incomplete	9 (5,7)
	Failure	62 (39,2)
**Last status**	Alive	50 (31,6)
	Exitus	108 (68,4)

**CNS.:** Central nervous system**, non-APL AML:** Non-acute promyelocytic leukemia.

There was a statistically significant relationship between the final status of the patients and initial age, presence of extramedullary disease at initial diagnosis, and response to induction therapy (*p* < 0.05, for all). The mortality rate was higher in those who were 60 years of age and above, who had extramedullary disease at initial diagnosis, and who were non-responder to induction therapy **(**Table 3**)**.

**Table 3:** The relationship between demographic characteristics of the patients and mortality.

		**Last status**		**X^2^**	**p**
		**Alive**	**Exitus**		
**Gender**	Female	24 (33,8)	47 (66,2)	0,126	**0,723**
	Male	26 (29,9)	61 (70,1)		
**Age**	<60	40 (40,8)	58 (59,2)	8948	0,003*
	≥60	10 (16,7)	50 (83,3)		
**Initial leukocyte count (/mm^3^)**	<50.000	33 (30,6)	75 (69,4)	0,062	**0,803**
	≥50.000	17 (34)	33 (66)		
**CNS involvement**	(-)	49 (33,1)	99 (66,9)	Fisher exact	**0,172**
	(+)	1 (10)	9 (90)		
**Extramedullary disease**	(-)	48 (38,7)	76 (61,3)	11,819	0,001*
	(+)	2 (5,9)	32 (94,1)		
**Presence of tumor lysis**	(-)	50 (31,8)	107 (68,2)	Fisher exact	**0,999**
	(+)	0 (0)	1 (100)		
**Risk stratification by genetic in non-APL AML**	Favorable	18 (27,3)	48 (72,7)	1367	**0,515**
	Intermediate	21 (32,8)	43 (67,2)		
	Poor	11 (39,3)	17 (60,7)		
**CD7**	(-)	29 (36,7)	50 (63,3)	1873	**0,171**
	(+)	21 (26,6)	58 (73,4)		
**CD34**	(-)	23 (41,8)	32 (58,2)	3347	**0,067**
	(+)	27 (26,2)	76 (73,8)		
**HLA DR...........**	(-)	8 (38,1)	13 (61,9)	0,185	**0,667**
	(+)	42 (30,7)	95 (69,3)		
**Response to induction therapy**	Responder	46 (52,9)	41 (47,1)	38,178	0,000*
	Non-responder	4 (5,6)	67 (94,4)		

**CNS.:** Central nervous system**, non-APL AML:** Non-acute promyelocytic leukemia.

There was a statistically significant relationship between the response status to induction therapy and the age at diagnosis, and CD34 positivity (*p* < 0.05, for all). The rate of non-responders to induction therapy was higher in those who were 60 years of age and above at initial diagnosis and those with CD34 positivity **(**Table 4**)**.

**Table 4:** The relationship between demographic characteristics and response status of the patients.

		**Response to induction therapy**		**X^2^**	**p**
		**Complete**	**Failure**		
**Gender**	Female	44 (62)	27 (38)	2487	**0,115**
	Male	43 (49,4)	44 (50,6)		
**Age**	<60	69 (70,4)	29 (29,6)	24,558	0,000*
	≥60	18 (30)	42 (70)		
**Initial leukocyte count (/mm^3^)**	<50.000	57 (52,8)	51 (47,2)	0,458	**0,498**
	≥50.000	30 (60)	20 (40)		
**CNS involvement**	(-)	79 (53,4)	69 (46,6)	1715	**0,190**
	(+)	8 (80)	2 (20)		
**Extramedullary disease**	(-)	72 (58,1)	52 (41,9)	1572	**0,210**
	(+)	15 (44,1)	19 (55,9)		
**Presence of tumor lysis**	(-)	86 (54,8)	71 (45,2)	Fisher exact	**0,999**
	(+)	1 (100)	0 (0)		
**Risk stratification by genetic in non-APL AML**	Favorable	36 (54,5)	30 (45,5)	1304	**0,521**
	Intermediate	33 (51,6)	31 (48,4)		
	Poor	18 (64,3)	10 (35,7)		
**CD7**	(-)	49 (62)	30 (38)	3095	**0,079**
	(+)	38 (48,1)	41 (51,9)		
**CD34**	(-)	37 (67,3)	18 (32,7)	4354	0,037*
	(+)	50 (48,5)	53 (51,5)		
**HLA DR...........**	(-)	10 (47,6)	11 (52,4)	0,251	**0,616**
	(+)	77 (56,2)	60 (43,8)		

**CNS.:** Central nervous system**, non-APL AML:** Non-acute promyelocytic leukemia.

**Fig. 2:**
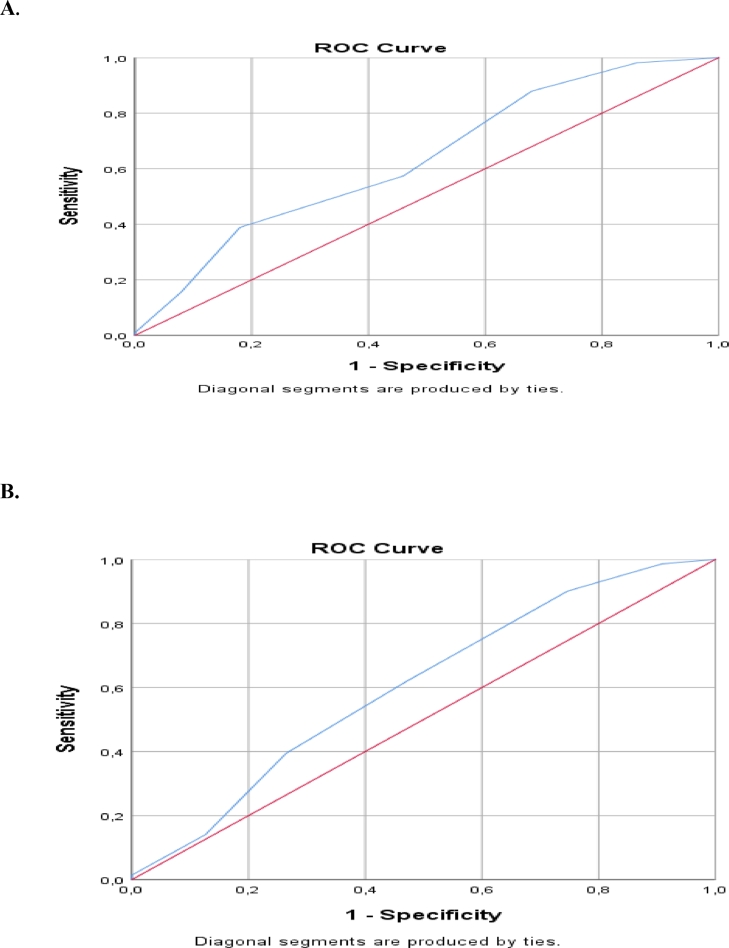
(A) ROC curve for mortality, (B) ROC curve for response status.

Effect of the risk scoring system on patients’ mortality and responsiveness to induction therapy was examined and statistically significant results were obtained (*p* < 0,05). The median value of risk score was determined as 2,5 points. The area under the curve (AUC) for the median risk score of 2,5 in ROC analysis was 0,635 (0,541–0,729; 95% confidence interval, *p* = 0,006) for exitus; while it was 0,605 (0,517–0,692; 95% confidence interval, *p* = 0,024) for being responder (Table 5**.,** Fig. 2**.**). The sensitivity for mortality was 88%, the specificity was 42%, the positive predictive value (PPV) was 90,1%, and the negative predictive value (NPV) was 24,7%. In terms of being non-responder to induction therapy, the sensitivity was 90,1%, the specificity was 25,3%, the PPV was 89,8%, and the NPV was 32% (Table 6**.**).

**Table 5:** ROC analysis of risk score for mortality and response status.

	**AUC**	**S.E.**	**p**	**Confidence interval 95%**	
				**Lower**	**Upper**
**Mortality**	0,635	0,048	0,006*	0,541	0,729
**Response to induction therapy**	0,605	0,045	0,024*	0,517	0,692

**AUC.:** Area under the curve**, S.E:** Standard error.

**Table 6:** Cut-off values and estimation probabilities of risk score for mortality and response status.

	**Sensitivity**	**Specificity**	**PP-**	**PP+**
**Mortality**	**0,880**	0,420	0,247	0,901
**Response to induction therapy**	**0,901**	0,253	0,320	0,898

There was a statistically significant difference between the patients who died and those who were alive in terms of risk score and overall survival (OS) (*p* < 0.05). While the median risk score was higher in patients who died (median: 4), OS was shorter (median: 5 months). There was a statistically significant difference in risk score and OS between responders and non-responders (*p* < 0,05). While the risk score was higher in non-responders (median: 4), OS was shorter (median: 3 months) (Table 7**.**).

**Table 7:** Comparison of OS and risk score by mortality and response status.

	**Last status**	**n**	**Mean±*s*.d.**	**Median**	**t**	**p**
Total risk score	Alive	50	3,26±1,45	3	−3098	0,002*
	Exitus	108	3,99±1,34	4		
OS (Months)	Alive	50	22,1 ± 20,53	12	4349	0,000*
	Exitus	108	8,78±10,16	5		
	**Response to induction therapy**					
Total risk score	Responder	87	3,52±1,48	3	−2417	0,017*
	Non-responder	71	4,06±1,29	4		
OS (Months)	Responder	87	18,78±17,56	12	6071	0,000*
	Non-responder	71	5,9 ± 8,25	3		

**OS.:** Overall survival, **s.d.:** Standard deviation.

**Fig. 3:**
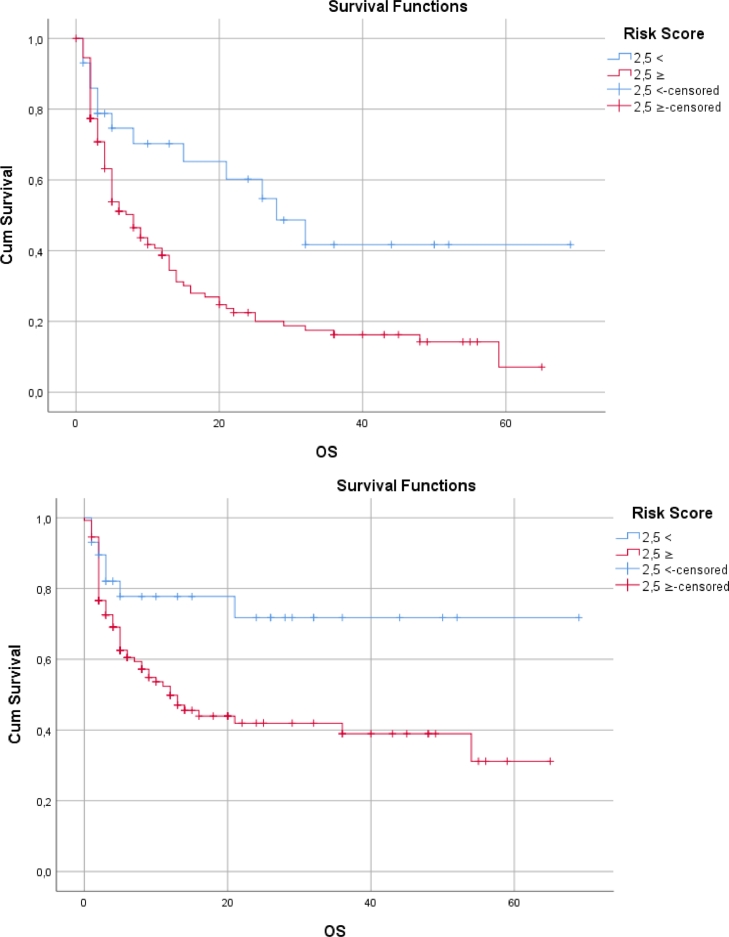
Kaplan-Meier Curves: (A) Survival by risk score for mortality, (B) Survival by risk score for response.

OS showed a statistically significant difference according to the risk factor score (*p* < 0,05). OS was shorter in those with high risk scores (Table 8**.,** Fig. 3**.**).

**Table 8:** Survival - Kaplan Meier analysis results.

		**Survival**	**S.E.**	**Confidence interval 95%**		**Log Rank**	**Breslow**	**Tarone-Ware**
				**Lower**	**Upper**			
Last status	<2,5	**36,917**	6130	24,903	48,931	0,004*	0,019*	0,007*
	≥2,5	**16,937**	2051	12,917	20,957			
Response to induction therapy	<2,5	**51,353**	5761	40,061	62,644	0,017*	**0,051**	0,030*
	≥2,5	**28,929**	3023	23,004	34,854			

**S.E:** Standard error.

## Discussion

This multi-factorial risk scoring system, which we developed for the use in AML prognosis prediction, provides both practical and highly effective clinical results. It has important findings in terms of combined use of flow cytometric features with clinical, cytogenetic/molecular results.

In the study of Ouyang et al. from 2019 [11], it was aimed to examine the association of different immunophenotypic features with leukemia survival. In the analysis performed in 470 AML patients, it was revealed that CD7, CD19, CD20 and HLA-DR........... positivity had a negative effect on OS. In another study from 2015 [9], flow cytometric immunophenotypic features were evaluated in a total of 142 patients with AML; CD7 expression has been shown to be a negative prognostic indicator. In this study, it was revealed that CD34 positivity is a positive prognostic marker. In another study [12], a total of 209 patients with AML were evaluated and the association of flow cytometric immunophenotyping with relapse was investigated. CD34 positivity, HLA-DR........... positivity, or a combination of both have been shown to be associated with relapse. In addition to the controversial literature data on CD34 positivity, our study showed that, CD34 positivity had a significant negative effect only on the induction response and did not have any effect on mortality of the patients. The association of aberrant CD7 positivity with Fms-like tyrosine kinase-3 internal tandem duplication (FLT3/ITD) mutation and poor prognosis has also been demonstrated [13]. At this point, it should be emphasized that the use of immunophenotypic features in combination with clinical and genetic features gains importance rather than using them alone. Considering both the sensitivity of immunophenotyping and the number of patients in the studies, it would be more reliable to develop new risk scoring systems.

Although the significant effect of flow cytometry on prognosis and treatment response has been examined in many studies before, its effectiveness and significance differs. In a study evaluating CD7 expression in AML cases [14], no association was found between CD7 aberrant expression and prognosis. In another study, CD7 and HLA-DR........... expression was not found to be significant in terms of CR, event free survival (EFS) and OS in univariate analysis; CD34 positivity showed a significant negative effect on EFS and OS [15]. When all these literature data are examined, it can be thought that flow cytometric features alone create controversial results, therefore it would be more meaningful to combine them with clinical, genetic and laboratory findings. In our study results, only CD34 positivity was found to be associated with response in univariate analysis; CD7 and HLA-DR........... positivity did not show any significance in terms of induction response and mortality.

There were important limitation points of our study. It had a limited patient population with a single-center data. Other surface antigens were not examined in the study because they were not evaluated in our clinic at the initial evaluation. Patients who had to be excluded from the study due to exclusion criteria also affect the final evaluation.

## Conclusion

As a result, the AUC for the median risk score of 2,5 in ROC analysis was 0,635 (0,541–0,729; 95% confidence interval, *p* = 0,006) for exitus; while it was 0,605 (0,517–0,692; 95% confidence interval, *p* = 0,024) for being responder. The sensitivity for mortality was 88%, the specificity was 42%, the PPV was 90,1%, and the NPV was 24,7%. In terms of being non-responder to induction therapy, the sensitivity was 90,1%, the specificity was 25,3%, the PPV was 89,8%, and the NPV was 32%. In addition to molecular and cytogenetic features to predict the prognosis in AML, this study makes an important contribution to the literature in terms of creating a different perspective including also clinical and flow cytometric features.

## Declarations

### Ethics approval and consent to participate

Ethical committee approval was received (Istanbul Training and Research Hospital, approval date and number: 13/11/2020, 2574) and the patients and control subjects gave informed consent before the beginning of the study. The experimental procedures were based on the Declaration of Helsinki and relevant institutional regulations.

### Patient consent for publication

Not applicable

### Availability of data and materials

The data that support the findings of this study are openly available: “SERIN, Istemi (2021), “Samatya Score”, Mendeley Data, V1, doi: 10.17632/jj9rvsct9w.1″

### Funding

No funding was received. None of the authors have disclosures relevant to this manuscript.

### Authors’ contributions

I.S. conceived the study; I.S., D.S., B.O., H.G., T.A.C., H.S., B.G., O.Y. acquired data; I.S. analyzed data; I.S., T.A.C., and H.G. wrote the original draft; all authors revised and approved the final manuscript.

## Declaration of Competing Interest

None to declare.
